# *Bifidobacterium animalis* KV9 and *Lactobacillus vaginalis* FN3 alleviated β-lactoglobulin-induced allergy by modulating dendritic cells in mice

**DOI:** 10.3389/fimmu.2022.992605

**Published:** 2022-09-15

**Authors:** Xiaoying Tian, Rongbo Fan, Hong He, Qingyu Cui, Xi Liang, Qiqi Liu, Tongjie Liu, Kai Lin, Zhe Zhang, Huaxi Yi, Piming Gong, Lanwei Zhang

**Affiliations:** ^1^ College of Food Science and Engineering, Ocean University of China, Qingdao, China; ^2^ College of Food Science and Engineering, Qingdao Agricultural University, Qingdao, China; ^3^ The Affiliated Hospital of Qingdao University, Qingdao, China

**Keywords:** anti-food allergy, β-lactoglobulin, probiotics, toll-like receptor 4, immunity

## Abstract

Food allergy is a serious public health problem because of its high incidence and risk. Probiotics can induce immune regulation in patients with allergic diseases, but its mechanism is not fully clear. In this paper, β-lactoglobulin (β-LG)-sensitized mice were used as models to explore the mechanism of *Bifidobacterium animalis* KV9 (KV9) and *Lactobacillus vaginalis* FN3 (FN3) on reducing allergic reactions and regulating immune cell function. The results showed that oral administration of KV9 and FN3 significantly reduced the scores of allergic symptoms, hypothermia symptoms, and serum levels of β-LG-specific immunoglobulins E (β-LG-sIgE), histamine, and mast cell protease in allergic mice. Flow cytometry analysis of intestinal dendritic cells (DCs) showed that the proportion of CD11c+major histocompatibility complex (MHC)-II+DCs, CD11c+CD80+DCs, and CD11c+ CD86+DCs increased after KV9 and FN3 intervention, indicating that the strains induced immature DCs and decreased the antigen-presenting capacity of DCs. Meanwhile, the toll-like receptor 4 (TLR4)-NF-κB signaling pathway was activated in DCs. The secretion of interleukin-12 (IL-12) was significantly increased, while interleukin-4 (IL-4) was decreased by DCs after KV9 and FN3 intervention, indicating that DCs have the potential to promote T-cell differentiation into T helper type 1 (Th1) cells. Furthermore, the proportion of CD3+CD8−IFN-γ+ T cells in the spleen increased, while CD3+CD8−IL-4+T cells decreased after oral administration of KV9 and FN3, correcting the T helper type 2 (Th2)-skewed immune responses. These results indicate that KV9 and FN3 reduce β-LG-induced allergic symptoms in mice, and suggest that the two potential probiotics might be used as an alternative therapeutic agent for mitigating food allergy.

## Introduction

The incidence of food allergy has increased rapidly in recent years, affecting nearly 5% of adults and 8% of children worldwide (1). Milk, egg, peanut, tree nuts, soy, wheat, fish, and crustacean shellfish are considered the eight major allergenic foods (2). Milk allergy is an allergic reaction caused by allergenic proteins in milk and dairy products, β-lactoglobulin (β-LG) is one of the most important allergens of the whey fraction, and accounts for 10% of total milk proteins (3). In addition, approximately 62% of cows’ milk allergy patients are sensitive to β-LG (4). Common food allergy is mediated by immunoglobulin E (IgE), which involves the uptake of antigens by antigen-presenting cells (APCs), such as dendritic cells (DCs), across the intestinal barrier. IgE intermediated by the binding of multivalent antigens at the surface of mast cells triggers the release of many allergy-related mediators such as histamine and leukotrienes. The production of IgE is affected by T helper type 1 (Th1)/T helper type 2 (Th2) cell balance (5). Treatment targeting the Th1/Th2 balance has become an effective approach. The most effective way to prevent food allergy is to strictly avoid food allergens; however, accidental ingestion is difficult to be absolutely avoided in our life. The first-line treatment is intramuscular epinephrine after the diagnosis of anaphylaxis. Epinephrine can maintain blood pressure, improve respirations, and decrease edema that may be causing airway collapse (6). H1 antihistamines are the next treatment that should be used for anaphylactic symptoms. As allergic reactions can cause histamine release, giving medications that block the H1 and H2 receptors can improve the vasculature integrity and maintenance of the blood pressure and heart rate (7). Medicine treatment only temporarily alleviates allergic symptoms and the suffering of anaphylaxis and cannot prevent the occurrence of food allergy in advance (8). Therefore, a new type of food allergy therapy is urgently needed.

Probiotics are live microorganisms that confer a health benefit to the host when administered in adequate amounts (9), and may be a safe alternative due to their capacity to affect the innate and adaptive immune system (10). There have been several studies showing that oral administration of probiotics could alleviate food allergy. Shandilya et al. reported that *Lactobacillus acidophilus* LaVK2 and *Bifidobacterium bifidum* BbVK3 reduced whey protein-induced intestinal anaphylaxis in mice by reversing the Th1/Th2 immune imbalance (11). In another study, *Lactobacillus rhamnosus* LA305, *Lactobacillus salivarius* LA307, and *Bifidobacterium longum* subsp. *Infantis* LA308 could contribute to alterations in immune responses in a mouse model of β-LG allergy, as well as anergy that could contribute to oral tolerance acquisition (12). DCs are commanders-in-chief of the immune system, in charge of the mechanisms of immune response/tolerance in the gut (13). In a previous study, bone marrow-derived mouse DCs were exposed to irradiated lethal lactic acid bacteria, and the results showed that all strains upregulated major histocompatibility complex (MHC)-II and B7-2 (CD86) on the surface of DCs, with *Lactobacillus casei* strongly inducing IL-12 secretion and *Lactobacillus reuteri* inducing IL-10 secretion, stimulating T cells to polarize into Th1 and Treg in the intestinal tract (14). Current studies have shown that probiotics can regulate immune function through DCs; nevertheless, the immunoregulation of DCs by probiotics is strain dependent and the knowledge of molecular mechanisms underlying probiotics–host interactions through DCs is still incomplete.

This study aimed to assess the effects of KV9 and FN3 on relieving β-LG-induced food allergy and to explore their potential mechanisms of action. We found that KV9 and FN3 alleviated the symptoms caused by food allergy and regulated the development and function of DCs and T cells. These results provide further insight into probiotics as a new type of food allergy therapy.

## Materials and methods

### The strains and preparation of bacterial suspension

*Bifidobacterium animalis* KV9 (KV9) and *Lactobacillus vaginalis* FN3 (FN3) were stored in the Functional Dairy and Probiotic Engineering Laboratory of Ocean University of China. The strains were cultured successively twice in de Man, Rogosa, and Sharpe (MRS) broth (Qingdao Hope Bio-Technology Co., Ltd., China) before use, then incubated anaerobically at 37°C for 24 h. The bacteria were washed twice by sterile phosphate-buffered saline (PBS) with pH 7.2 and collected by centrifugation and resuspended in PBS to give a concentration of 5×10^8^ colony-forming units (CFU)/ml.

### Animals and construction of food allergy model

Balb/c mice (female, 5 weeks old) were purchased from Beijing Vital River Laboratory Animal Technology Co., Ltd. China. Mice were kept in plastic cages and allowed free access to diet (Beijing Keao Xieli Feed Co., Ltd., China) and water, animal rooms were maintained on a 12-h light/dark cycle, and the air was exchanged at 15 times/h with room temperature and humidity of 22 ± 1°C and 55 ± 10%, respectively. All experimental processes were approved by the Animal Ethics Committee of Ocean University of China (permission number: SPXY2021112401).

Mice were acclimatized to the animal facility for 1 week before experimentation. Briefly, the mice were divided randomly into the following groups (*n* = 10 per groups): non-sensitized (control), β-LG-sensitized and challenged (allergy), hydrocortisone supplement and β-LG-sensitized and challenged (medicine), KV9 supplement and β-LG-sensitized and challenged (KV9), and FN3 supplement and β-LG-sensitized and challenged (FN3). Mice were sensitized according to previous research and modified by intraperitoneal injection of 50 μg of β-LG (Sigma Aldrich Co., LTD, USA) and 100 μg of Imject™ Alum Adjuvant (Alum, Thermo Fisher Scientific Co., LTD., USA) in 200 μl of sterile PBS on day 14, and boosted with 100 μg of β-LG and 100 μg of Alum on day 28 in 200 μl of sterile PBS (15). Bacterial suspension (0.2 ml, containing 10^8^ live bacteria) and PBS (0.2 ml) were administered *via* oral gavage to β-LG-sensitized mice from day 7 to day 52 in the KV9 groups, FN3 groups, and control groups, respectively (16). Hydrocortisone (0.5 mg/kg bw, Aladdin Reagent Co., LTD., China) in 200 μl of PBS was administered *via* oral gavage to β-LG-sensitized mice from day 28 to day 52 in the medicine group. Mice were orally challenged six times with 5 mg of β-LG in sterile PBS every 2 days from day 42. Before challenge, mice were deprived of food for 2 h.

### Evaluation of allergic reaction in animals

The core body temperatures were monitored every 15 min for 1 h after the β-LG challenge, and the temperature usually decreased when an allergy occurs. Allergic symptoms were monitored for 15 min and scored as follows (17): 0—no signs; 1—mice are scratching between 4 and 10 times for 15 min; 2—mice are scratching more than 10 times for 15 min, or display reduced activity or bristled fur; 3—mice have a strongly reduced activity, display liquid diarrhea, and have difficulty in walking normally, bristled fur, and sometimes labored respiration; 4—manifestations of degree 3 are stronger, and mice displayed cyanosis around the mouth and tail; 5—death.

### Measurement of serum cytokine and β-LG-specific IgE

Blood was obtained 1 h after the final challenge, and serum was obtained by centrifugation. Serum levels of histamine, mMCPT-1, and β-LG-specific IgE (β-LG-sIgE) were determined using an ELISA kit (Nanjing Jiancheng Technology Co., Ltd., China) according to the instructions.

### Culture of mouse lymphocytes and DCs

Spleen and intestinal lymph nodes were extracted from mice under aseptic conditions, cut into pieces, and ground on a 70-μm cell filter. Mouse lymphocytes and DCs were isolated by the Mouse Splenic Lymphocyte Isolation Kit (Beijing Solarbio Science & Technology Co., Ltd., China) and the Mouse Tissue Dendritic Cell Isolation Kit (Tianjin Haoyang Biological Manufacture Co., Ltd, China), respectively. The regulated cell concentration of 5 × 10^6^ cells/ml was suspended in Roswell Park Memorial Institute (RPMI-1640, Corning Incorporated Co., Ltd., China) medium with 10% fetal bovine serum (FBS, Biological Industries Israel Beit Haemek Co., Ltd., Israel), 100 units/ml penicillin (Beijing Solarbio Science & Technology Co., Ltd., China), and 100 μg/ml streptomycin (Beijing Solarbio Science & Technology Co., Ltd., China), cultured for 3 days (18). IFN-γ and IL-4 from lymphocyte culture supernatants and IL-12 and IL-4 from DC culture supernatants were detected using an ELISA kit according to the manufacturer’s instructions (Nanjing Jiancheng Technology Co., Ltd., China).

### Flow cytometry analysis of DCs and T cells

Single cells isolated from spleen and intestinal lymph node were stained for FACS analysis as described previously (16). Lymphocytes were stained using the Zombie Violet™ Fixable Viability Kit (BioLegend Biotechnology Co., LTD, China) and were stained for surface markers including CD3-APC-Cy7 and CD8-APC (Thermo Fisher Scientific Co., LTD., USA); cells were then fixed and permeabilized by the Intracellular Fixation and Permeabilization Buffer Set (Thermo Fisher Scientific Co., LTD., USA) and stained for intracellular expression markers such as IFN-γ-PerCP and IL-4-PE (Thermo Fisher Scientific Co., LTD., USA). DCs were stained for surface markers including CD11c+-PE-Cy7, CD80-APC, CD86-FITC, and MHC-II-PE (Thermo Fisher Scientific Co., LTD., USA). Data were acquired with BD FACSVerse (Becton Dickinson Co., LTD., Canada) and analyzed with BD FACSuite (Becton Dickinson Co., LTD., Canada).

### Gene expression determination by qPCR

DCs were employed to examine the toll-like receptor 4 (TLR4)-NF-κB relative expression of genes (19). The total RNA was extracted from spleens using Trizol Reagent (Tiangen Biotech CO., LTD., China), and total RNA was reverse transcribed using the ReverTra Ace qPCR RT Master Mix with gDNA Remover Kit (Toyobo CO., LTD., Japan). Quantitative PCR was performed using the SYBR Green Realtime PCR Master Mix (Toyobo CO., LTD., Japan) according to the instructions on a CFX96 Real-Time System (Bio-Rad Laboratories CO., LTD., USA). For qPCR, the reaction mixture contained 8 μl of ddH_2_O, 12 μl of SYBR qPCR Mix, 2.5 μl of forward primer and reverse primer, and 2.5 μl of complementary DNA. The conditions for qPCR were as follows: 95°C for 60 s, followed by 40 cycles of 95°C for 15 s, 60°C for 15 s, and 72°C for 45 s. The 2^−ΔΔCT^ method was used to calculate the relative mRNA levels, and β-actin was used as the internal control. Primers for qPCR were designed and synthesized by Sangon Biotech (Shanghai) Co., Ltd (China). The primers are shown in **Table 1**.

**Table 1:** RT-PCR amplified primers.

Gene name	Forward (5’-3’)	Reverse (5’-3’)
β-actin (M)	CTGTCCCTGTATGCCTCTG	ATGTCACGCACGATTTCC
TLR4	GGCATGGCTTACACCACCTC	TTGTCTCCACAGCCACCAGA
Myd88	AGCAGAACCAGGAGTCCGAGAAG	GGGCAGTAGCAGATAAAGGCATCG
TRAF6	GACTGCCCAACAGCTCCAATCC	AAGTGTCGTGCCAAGTGATTCCTC
IκB	TGGTGTGACTGTGGATCTCTGGAG	GGCTGGCTTCTCTGTGGTGATTC
NF-κB	GGATATGAGGAAGCGGCATGTAGAG	CCTGATACTGGCACTTCGGACAAC
IL-12	TCTTTGATGATGACCCTGTGCCTTG	GTGATTCTGAAGTGCTGCGTTGATG
IL-4	TACCAGGAGCCATATCCACGGATG	TGTGGTGTTCTTCGTTGCTGTGAG

### Statistical analysis

All data were expressed as the mean ± SD. Statistical differences between the two groups were analyzed using Student’s *t*-test; *p* values <0.05 were considered significant and are indicated by asterisks (*). Statistical differences between three or more groups were analyzed using a one-way analysis of variance and Duncan test for multiple comparisons by the Statistical Package for the Social Sciences 25 (SPSS 25), and the lack of significance was indicated by the same letters.

## Results

### Effect of oral probiotics on suppressing allergic sensitization of β-LG

**Figure 1:**
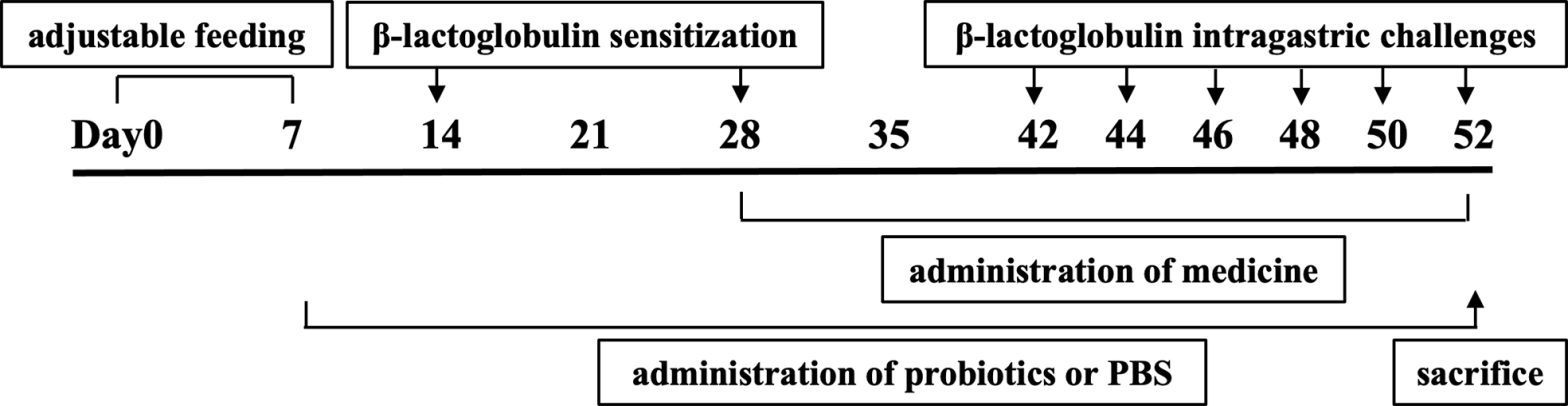
Protocol of mice sensitization and challenge.

**Figure 2:**
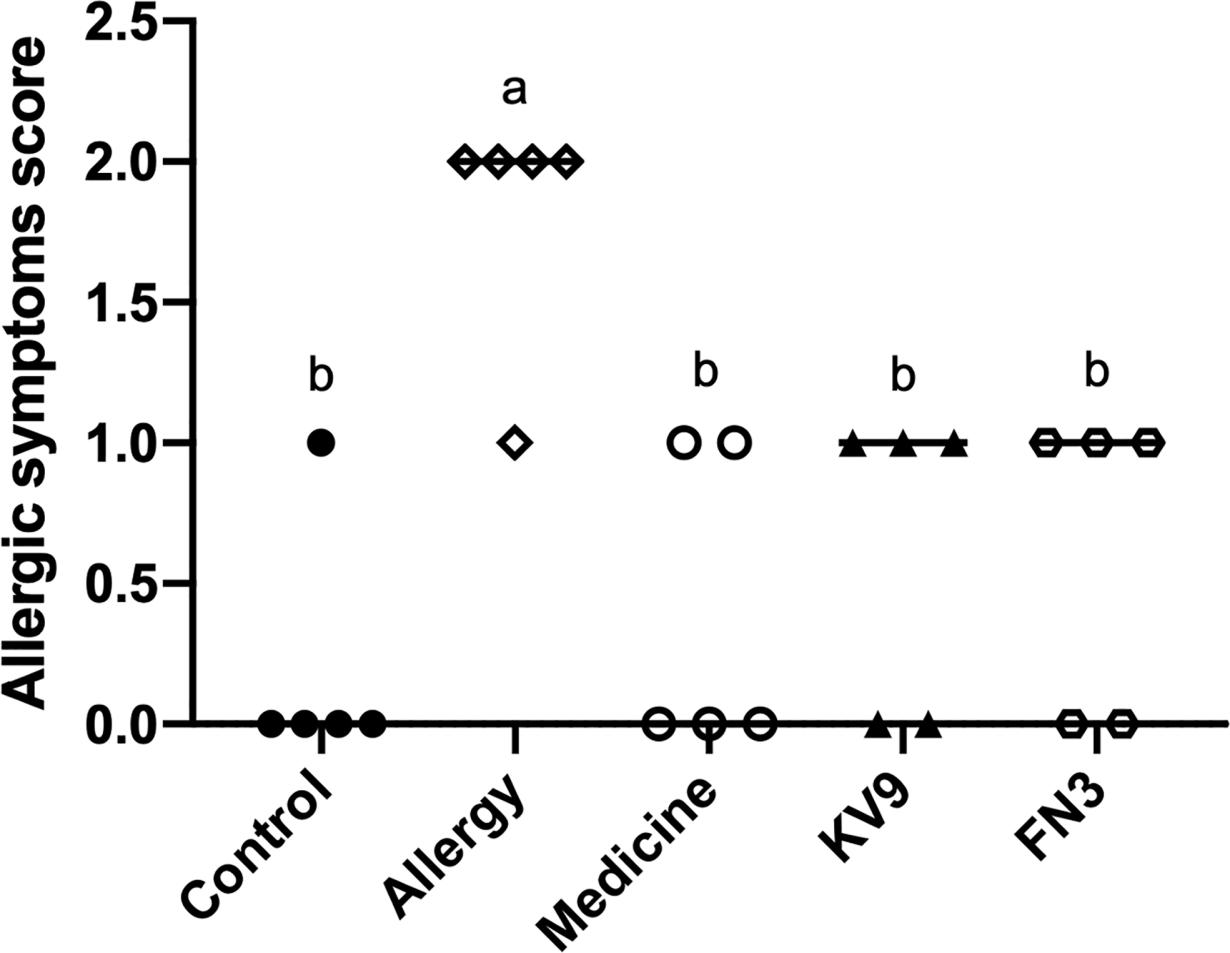
Allergic symptom scores in mice. Identical lowercase in the figure indicates the lack of significance (*p* < 0.05).

**Figure 3:**
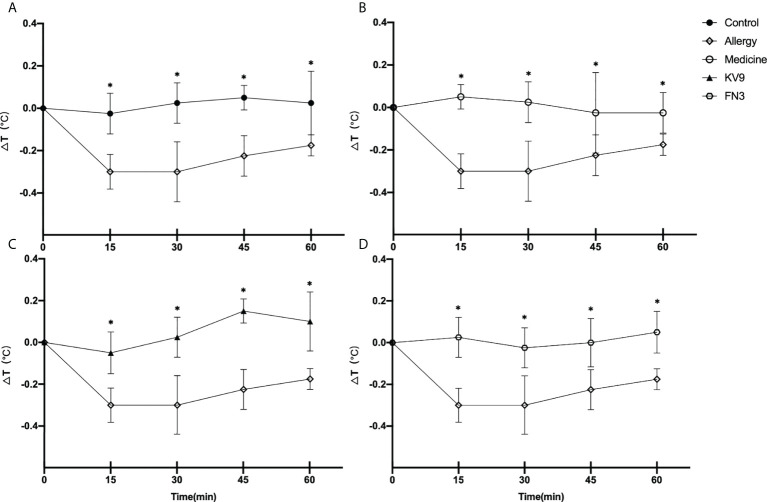
Changes in core body temperature at indicated time points following first challenge with β-LG in control **(A)**, medicine **(B)**, KV9 **(C)**, and FN3 **(D)** group mice compared with mice in the allergy group (*p < 0.05).

**Figure 4:**
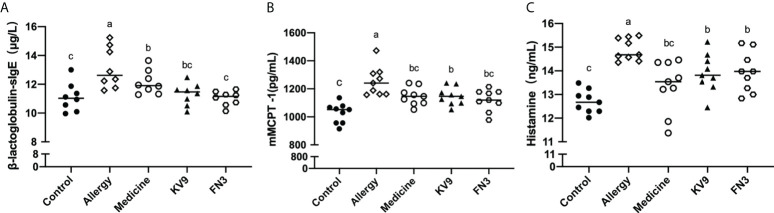
β-LG-sIgE **(A)** in the serum of mice. mMCPT-1 **(B)** in the serum of mice. Histamine **(C)** in the serum of mice. Identical lowercase in the figure indicates the lack of significance (*p* < 0.05).

In order to evaluate the effect of oral probiotics on allergic reactions in mice, we established a food allergy model in mice by intraperitoneal injection of β-LG; the protocol is shown in **Figure 1**. In allergic animals, continuous oral β-LG stimulation can cause a significantly higher allergic symptoms score, and the specific manifestation was scratching and bristling fur (**Figure 2**). At the same time, the core body temperatures of allergic animals decreased significantly (**Figure 3**). Oral administration of KV9 and FN3 led to a significant attenuation of allergic symptoms score 15 min after the final oral β-LG challenge with similar ability and no significant difference with the control group (**Figure 2**). Meanwhile, the core body temperature in allergic mice was reduced, and oral administration of KV9 and FN3 significantly alleviated hypothermia in allergic mice (**Figure 3**). The serum levels of β-LG-sIgE, histamine, and mMCPT-1 (**Figure 4**) in the allergic mice were higher than those of control mice after repetitive challenges, indicating severe allergic reactions in mice. The high levels of β-LG-sIgE were decreased by KV9 and FN3 supplementation and have no significant difference from the control group; the FN3 group was slightly better than the medicine group. The serum levels of mMCPT-1 and histamine were decreased by KV9 and FN3 supplementation in allergic mice. However, histamine levels in the KV9 and FN3 groups did not return to the same level as in the control group. These results confirm that the probiotics supplementation suppresses allergic sensitization to β-LG.

### Effect of oral probiotics on regulating DCs’ maturation and function

**Figure 5:**
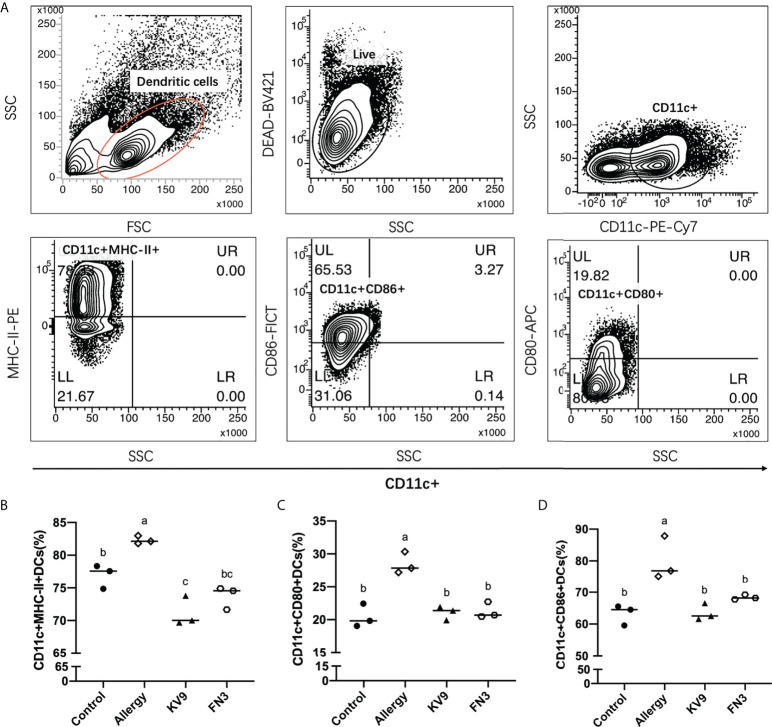
The effect of probiotics on DCs. The gating strategy **(A)** and the effect of oral administration of KV9 and FN3 on populations of CD11c+MHC-II+DCs **(B)**, CD11c+CD80+DCs **(C)**, and CD11c+CD86+DCs **(D)** in the intestinal lymph node from different groups of mice. Identical lowercase in the figure indicates the lack of significance (*p* < 0.05).

**Figure 6:**
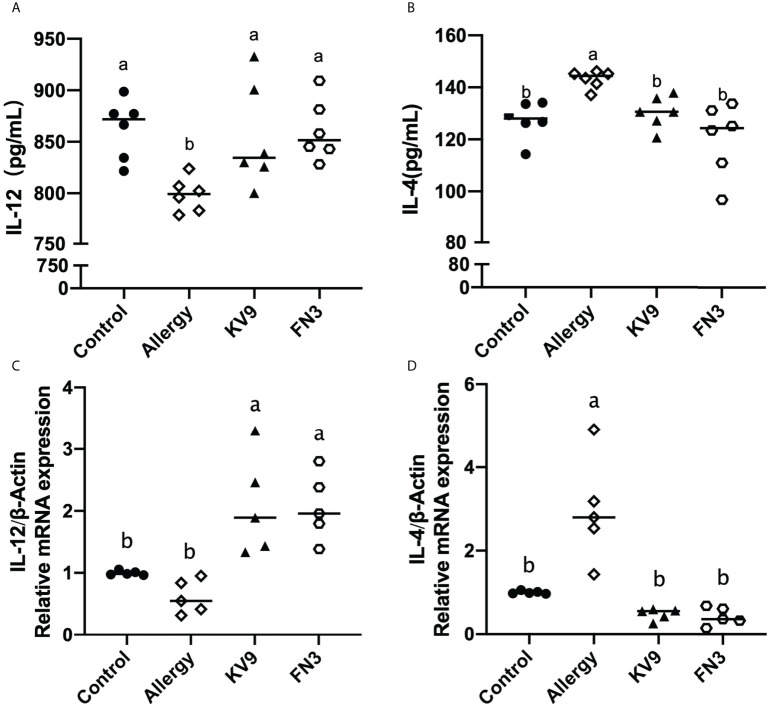
The IL-12 **(A)** and IL-4 **(B)** cytokine levels secreted from DCs and gene expression of IL-12 **(C)** and IL-4 **(D)** in DCs of each group of mice. Identical lowercase in the figure indicates the lack of significance (*p* < 0.05).

Allergic reactions to food begin with the uptake of food allergens by DCs, processed and presented to T cells. The ability of activated DCs to prime T-cell activity is attributable to DCs’ expression of MHC-II and costimulatory molecules that are upregulated during maturation (20). The expression of maturation of DCs was analyzed to investigate the possible functional relevance of DCs to probiotics suppressing allergic sensitization. FACS analysis showed that oral administration of KV9 and FN3 significantly reduced the percentage of CD11c+MHC-II+DCs, CD11c+CD80+DCs, and CD11c+CD86+DCs in CD11c+DCs (**Figure 5**). This indicated that the probiotics induced immature DCs. In order to gain insight into the effect of the administration of probiotics to allergic mice on expression patterns of cytokine production in DCs, cytokine production and gene expression were analyzed by DCs purified from the mice after the β-LG challenge. Oral β-LG significantly increased the *in vitro* IL-4 production and reduced the IL-12 by DCs (**Figure 6**). However, there was no significant difference in IL-4 and IL-12 production by DCs between the mice supplemented with probiotics and mice in the control group. The IL-4 and IL-12 gene expression confirmed the results (**Figure 6**).

### Effect of oral probiotics on activating Toll-like receptor 4-NF-κB

**Figure 7:**
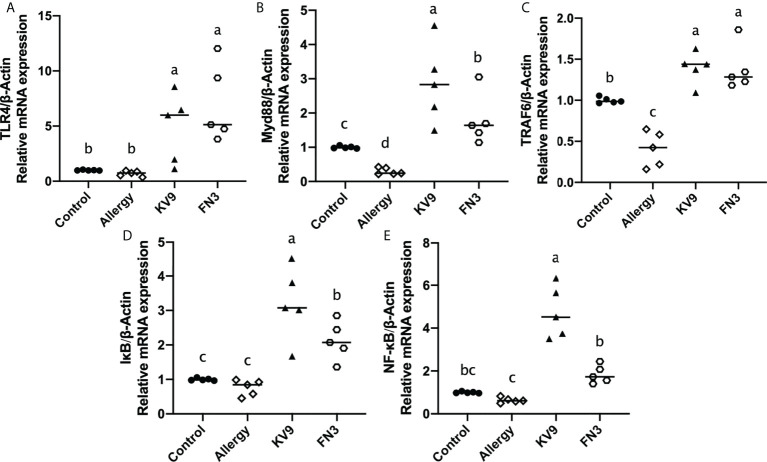
Gene expression of the TLR4-NF-κB signal pathway in DCs. The results were quantified as the density ratio between the gene of interest and the reference standard (β-actin). Identical lowercase in the figure indicates the lack of significance (*p* < 0.05).

TLR4 is a class of pattern recognition receptors that recognize the probiotics and activate adaptive immunity, which activates downstream signals when they bind to specific ligands. To explore the effect of the probiotics on TLR4 and downstream signal gene expression of DCs, gene expression of TLR4, Myd88, TRAF6, IκB, and NF-κB was detected. Compared with control mice, the expression of TLR4, Myd88, TRAF6, IκB, and NF-κB genes (**Figure 7**) was significantly increased after KV9 and FN3 supplementation; thus, the probiotics indicate activation of the TLR4-NF-κB signaling pathway.

### Effect of oral probiotics on regulating T-cell differentiation and function

**Figure 8:**
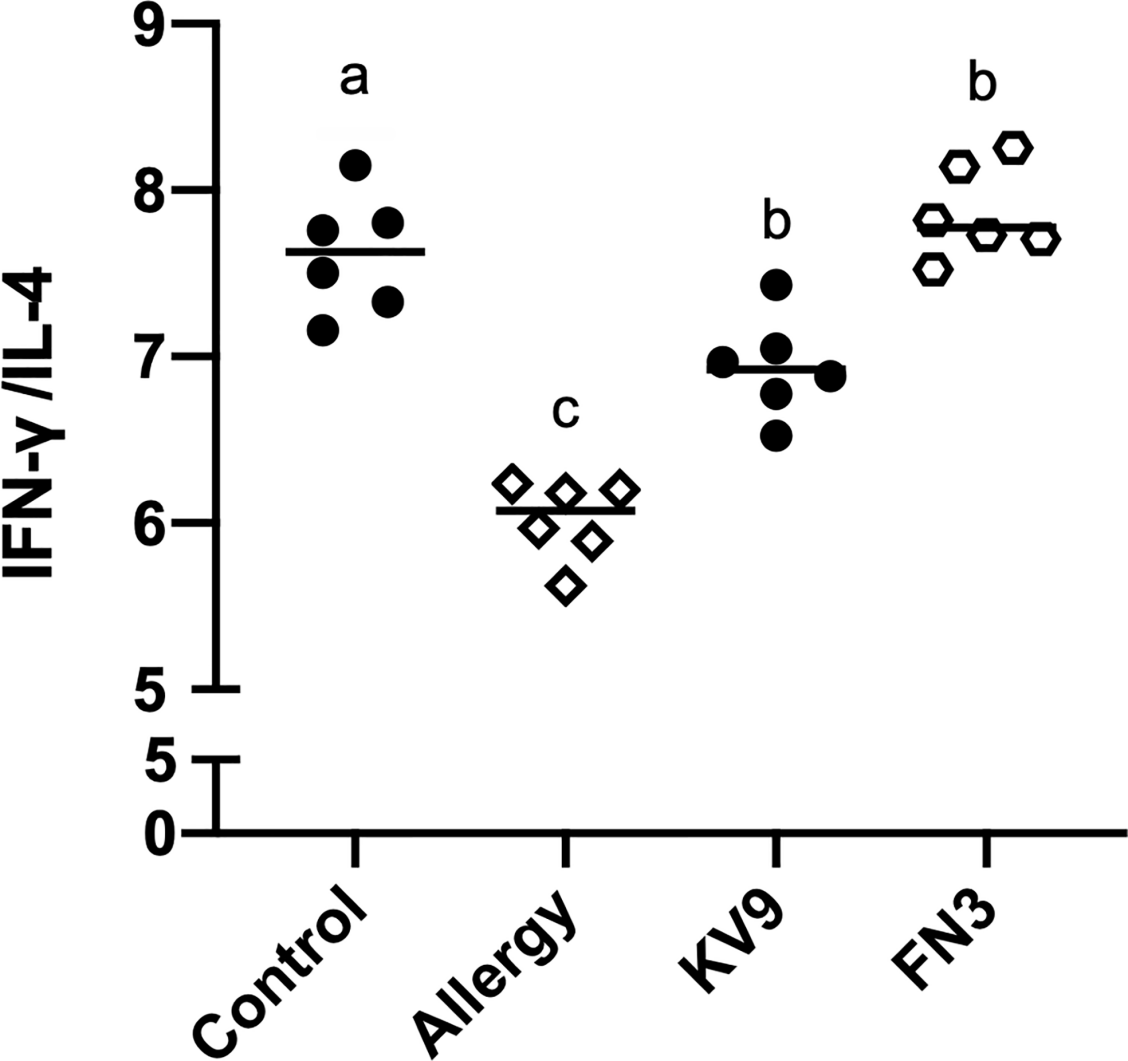
The ratio of IFN-γ (pg/ml)/IL-4 (pg/ml) cytokine levels secreted from spleen lymphocytes. Identical lowercase in the figure indicates the lack of significance (*p* < 0.05).

**Figure 9:**
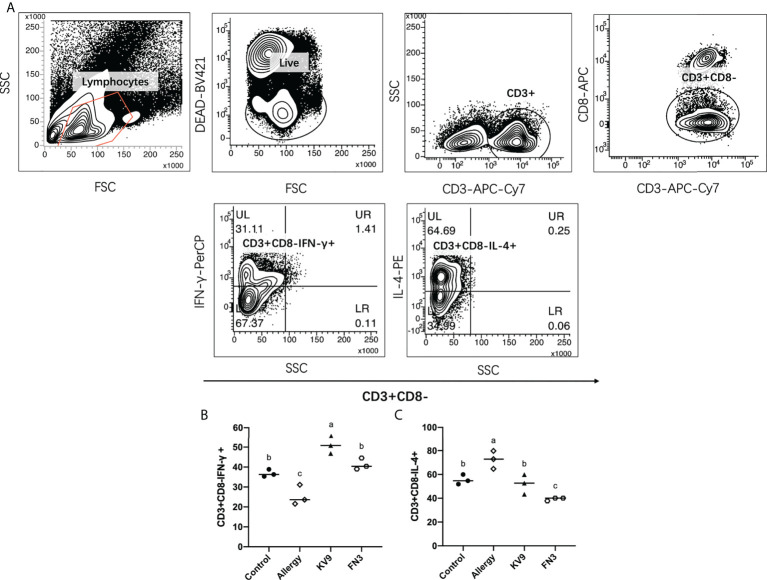
The effect of probiotics on T cells. The gating strategy **(A)** and the effect of oral administration of KV9 and FN3 on populations of CD3+CD8−IFN-γ+ T cells **(B)** and CD3+CD8−IL-4+ T cells **(C)** in the spleen from different groups of mice. Identical lowercase in the figure indicates the lack of significance (*p* < 0.05).

T cells play an important role in the food allergy process, especially in the Th1/Th2 immune balance (21). To investigate whether T-cell function changed after ingestion of the probiotics, spleen cells were isolated from mice after the last oral challenge, and their ability to produce Th1-related cytokine IFN-γ and Th2-related cytokine IL-4 *in vitro* was tested. Compared with control mice, the proportion of IFN-γ/IL-4 (**Figure 8**) in a splenic cell culture supernatant of allergic mice was significantly reduced. Oral administration of KV9 and FN3 increased the proportion of IFN-γ/IL-4 significantly compared with the allergic mice, but differed from mice in the control group. To directly evaluate the T-cell differentiation regulated by the probiotics, the percentage of Th1 cells and Th2 cells was measured by FACS (**Figure 9**). IFN-γ and IL-4 need to be stained after the Intracellular Fixation and Permeabilization Buffer Set treatment, which affects the expression of CD4+; thus, this expressed CD4+ gate by CD3+CD8−. The results showed that CD3+CD8−IL-4+T cells were predominant in the spleen of allergic mice and the proportion of CD3+CD8−IL-4+T cells in the spleen of KV9 and FN3 supplement mice decreased while CD3+CD8−IFN-γ+T cells increased, confirming that the probiotics skewed the Th1/Th2 immune balance toward Th1 and thus suppressed downstream allergic reactions. This is similar to the result of exocrine cytokines from the cell body.

## Discussion

The number of patients diagnosed with food allergies has been increasing in many countries. Evidence has shown that probiotics can exert a variety of probiotic effects by adhering to intestinal endothelial cells, producing antibacterial metabolites, competing with pathogenic microorganisms for nutrition, strengthening the epithelial barrier, changing the immune response pathway, and regulating the immune system (22, 23). In recent studies, probiotics such as *B. longum* and *Lactobacillus plantarum* have shown antiallergic potential in both animal models (12, 24). In the previous study of our laboratory, six probiotics capable of regulating the secretion of IFN-γ/IL-4 by peripheral blood mononuclear cells were screened out from 48 strains. Through the food allergy mouse model, two probiotics with good ability to alleviate food allergy were selected, namely, KV9 and FN3. In this study, oral administration of KV9 and FN3 reduced the score of allergic symptoms, the production of β-LG-sIgE in serum, the activation of mast cells, and the concentration of histamine in serum, and relieved the decrease of core body temperature, confirming that KV9 and FN3 were functional probiotics that can suppress allergy in mice. The exploration of the mechanism found that the probiotics induced the immature state of DCs, and significantly increased the secretion of IL-12 in mouse DCs through activating the TLR4-NF-κB signaling pathway; IL-12 is a central cytokine for the development of Th1 cells (25), resulting in the immune balance of T cells being skewed towards Th1-dominated direction.

Food allergy is often associated with Th2-skewed immune responses; IL-4 as a marker of Th2 cells promotes IgE production by B cells, while Th1 cells’ cytokine IFN-γ can inhibit Th2 cells’ function (5). Oral administration of KV9 and FN3 can reduce the level of β-LG-sIgE in serum of allergic mice; IgE is the main immunoglobulin in mast cell degranulation, indicating that the two probiotics play an immune regulatory role. The DCs are the most powerful APCs, the initiator and modulator of the immune response. DCs support and fine-tune antigen presentation through costimulatory molecules and cytokines. Depending on the conditions, DCs can stimulate the growth and activation of a variety of T cells, thereby having varying effects on the immune response (14). It is well known that the function of naïve T cells needs two specific signals from APC to be activated (26). The first specific signal is provided by the interaction between T-cell receptors and the major histocompatibility complex (MHC)-II on APC. The second signal is provided by the interaction between APC-provided costimulatory molecule ligands and CD28 and CTLA-4 receptors on T cells. Inhibition of costimulatory molecules on cell surface or intracellular signal transduction can induce T cells to have no response (27). The interaction between B7-1(CD80)/B7-2(CD86) on APC and CD28 determines whether T-cell reaction occurs (28). DCs have strong migration ability when they are immature, and DCs have strong antigen presentation ability when they are mature, which leads to a greater risk of food allergy (13). Furthermore, the expressions of DCs’ surface MHC-II and CD80/CD86 can be used as a marker of maturation (29). In the presence of mature DCs and the IL-12, they produce T cells that tend to become Th1 cells, which secreted IFN-γ, while the DCs induced T cells to differentiate into Th2 cells that secreted IL-4 and IL-5 under the action of IL-4 (13, 25). The expression of costimulatory molecules and cytokines in DCs depends on signals from their environment, including pathogen-associated molecular patterns (PAMPs), damage-associated molecular patterns, and cytokines (30). The key mechanism of probiotics-mediated immune regulation may be the regulation of DCs’ function. In a previous study, exposure of bone marrow-derived mouse DC to irradiated *L. casei* resulted in strong induction of IL-12 secretion, a potential strain that stimulates T cells to polarize into Th1 in the intestinal tract (14). The aforementioned study also revealed that *L. reuteri* can induce high expression of B7-2 (CD86) costimulatory in DC to polarize T cells into Th2 cells (14). Huang et al. confirm that oral *Lactobacillus murinus* suppressed the expression of OX40L in duodenal, a DC-derived costimulatory molecule known to promote Th2 responses, and indicates that DCs’ functions are modulated (31). Furthermore, Ma’s research on oral probiotics supplementation on DCs induced CD103+DCs’ expression, which promoted differentiation of FoxP3+Tregs (32). In this study, the expression levels of MHC-II, CD80, and CD86 decreased in the CD11c+DCs in the intestinal lymph nodes of each group of mice after oral KV9 and FN3. This implied that immature DCs increased and the antigen presentation ability of DC was positively correlated with its maturity (33). At the same time, the secretion of IL-12 in DCs significantly increased after oral administration of KV9 and FN3 compared with allergic mice.

IL-12 is a central cytokine in the activation of immunity in the generation of Th1-type responses; defective IL-12 production was associated with lack of NF-κB activation (34). A study revealed that mice with TLR4 deficiency after allergen stimulation showed food allergy and immune tendency to Th2 (35). The author observed that after killing the intestinal flora of both healthy mice and TLR4-deficient mice with antibiotics, the mice showed food allergy. In addition, the healthy mice showed oral tolerance to food antigens, while allergy reactions persisted in the TLR4-deficient mice, indicating that intestinal microbes play an important role in food tolerance through the TLR4 signaling pathway (35). However, C3H/HeJ mice with corrected TLR4 alleles were sensitized and challenged with peanuts in a study (36), revealing that mice were resistant to anaphylaxis after the oral peanut challenge; restoring TLR4 function in mice did not protect them from anaphylaxis. A similar conclusion was obtained in Berin’s study, which revealed that TLR4-deficient mice did not show Th2 immune advantage and food allergic reaction (37). Recent research suggested that TLR4, as an upstream target of the NF-κB pathway, plays an important role in oral tolerance to food antigens. In a study on *L.* casei, Zhang provided an intervention to achieve tolerant cell CD4+CD25+Treg differentiation by regulating NF-κB signal in which TLR2 and TLR4 were activated (16). Notably, in our research, the activation of TLR4-NF-κB signaling pathway by KV9 and FN3 in DCs resulted in the significant increase in the secretion of IL-12. This suggests that the supplementation of KV9 and FN3 enabled DCs to induce T cells to differentiate into Th1 cells.

In line with this notion, each group of mouse spleen lymphocytes in FACS analysis was extracted to determine whether oral KV9 and FN3 had an impact on allergic mice T-cell function and differentiation. The results showed a significantly lower proportion of Th1 cells in allergic mice spleen and a higher proportion of Th2 cells compared with mice in the control group. KV9 and FN3 supplementation increased the Th1 proportion, decreased the Th2 proportion significantly, and regulated T-cell cytokines of the IFN-γ/IL-4 secretion ratio in the supernatant of mouse spleen cells cultured *in vitro*. These results indicated that KV9 and FN3 alleviated the Th2 immune skew induced by food allergy.

In conclusion, our research demonstrated that *B. animalis* KV9 and *L. vaginalis* FN3 are potential probiotics against food allergy. The strains of KV9 and FN3 regulate DCs’ development and IL-12 production to promote Th1 immune and reduce allergic reactions in mice. These findings suggest that strains KV9 and FN3 may be used as candidate probiotics to prevent Th2-mediated allergic diseases. The functional substances of KV9 and FN3 to relieve food allergy will be studied in the future.

## Data availability statement

The raw data supporting the conclusions of this article will be made available by the authors, without undue reservation.

## Ethics statement

The animal study was reviewed and approved by Animal Ethics Committee of Ocean University of China.

## Author contributions

Conceptualization: LZ and PG. Methodology: XT, LZ, and PG. Formal analysis: XT, RF, and HH. Supervision: LZ and PG. Data curation: XT and QC. Writing—original draft preparation: XT, XL, HY, and QL. Writing—review and editing: TL, KL, and ZZ. Project administration: LZ. Funding acquisition: LZ. All authors contributed to the article and approved the submitted version.

## Funding

This work was supported by the Project of Taishan Industry Leading Talent of Shandong Province (LJNY202101) and the National Key R & D of China (2018YFC1604300).

## Conflict of interest

The authors declare that the research was conducted in the absence of any commercial or financial relationships that could be construed as a potential conflict of interest.

## Publisher’s note

All claims expressed in this article are solely those of the authors and do not necessarily represent those of their affiliated organizations, or those of the publisher, the editors and the reviewers. Any product that may be evaluated in this article, or claim that may be made by its manufacturer, is not guaranteed or endorsed by the publisher.

## References

[1] Lopes JP Sicherer S. Food allergy: Epidemiology, pathogenesis, diagnosis, prevention, and treatment. Curr Opin Immunol (2020) 66:57–64. doi: 10.1016/j.coi.2020.03.014 32446135

[2] Boyce JA Assa’ad A Burks AW Jones SM Sampson HA Wood RA. Guidelines for the diagnosis and management of food allergy in the united states: Report of the NIAID-sponsored expert panel preface. J Allergy Clin Immun (2010) 126(6):S1–58. doi: 10.1016/j.jaci.2010.10.007 21134576 PMC4241964

[3] Hochwallner H Schumeister U Swoboda I Spitzauer S Valenta R. Cow’s milk allergy: From allergens to new forms of diagnosis, therapy and prevention. Methods (2014) 66(1):22–33. doi: 10.1016/j.ymeth.2013.08.005 23954566 PMC3969108

[4] Spies J. Milk allergy. J Milk Food Technol (1973) 36:225–31. doi: 10.4315/0022-2747-36.4.225

[5] Anvari S Miller J Yeh CY Davis CM. IgE-mediated food allergy. Clin Rev Allerg Immu (2018) 57(2):244–60. doi: 10.1007/s12016-018-8710-3 30370459

[6] Sicherer SH Simons FER. Self-injectable epinephrine for first-aid management of anaphylaxis. Pediatrics (2007) 119(3):638–46. doi: 10.1542/peds.2006-3689 17332221

[7] Simons FER Simons KJ. Histamine and H1-antihistamines: Celebrating a century of progress. J Allergy Clin Immunol (2011) 128(6):1139–50. doi: 10.1016/j.jaci.2011.09.005 22035879

[8] Oyoshi M Chinthrajah RS. Editorial: Insights into the etiology, prevention, and treatment of food allergy. Front Immunol (2020) 11:1937. doi: 10.3389/fimmu.2020.01937 32973797 PMC7473206

[9] Kerry RG Patra JK Gouda S Park Y Shin HS Das G. Benefaction of probiotics for human health: A review. J Food Drug Anal (2018) 26(3):927–39. doi: 10.1016/j.jfda.2018.01.002 PMC9303019 29976412

[10] Rautava S Kalliomäki M Isolauri E. New therapeutic strategy for combating the increasing burden of allergic disease: Probiotics–a nutrition, allergy, mucosal immunology and intestinal microbiota (NAMI) research group report. J Allergy Clin Immun (2005) 116(1):31–7. doi: 10.1016/j.jaci.2005.02.010 15990769

[11] Shandilya UK Sharma A Kapila R Kansal VK. Probiotic dahi containing *Lactobacillus acidophilus* and *Bifidobacterium bifidum* modulates immunoglobulin levels and cytokines expression in whey proteins sensitised mice. J Sci Food Ag (2016) 96(9):3180–7. doi: 10.1002/jsfa.7497 26459934

[12] Esber N Mauras A Delannoy J Labellie C Waligora-Dupriet AJ Kashima T. Three candidate probiotic strains impact gut microbiota and induce anergy in mice with cow’s milk allergy. Appl Environ Microb (2020) 86(21):e01203–01220. doi: 10.1128/AEM.01203-20 PMC7580549 32826221

[13] Banchereau J Steinman RM. Dendritic cells and the control of immunity. Nature (1998) 392(6673):245–52. doi: 10.1038/32588 9521319

[14] Christensen HR Frokiaer H Pestka JJ. *Lactobacilli* differentially modulate expression of cytokines and maturation surface markers in murine dendritic cells. J Immunol (2002) 168(1):171–8. doi: 10.4049/jimmunol.168.1.171 11751960

[15] Fu GM Zhao K Chen H Wang YY Nie LJ Wei H. Effect of 3 *Lactobacilli* on immunoregulation and intestinal microbiota in a β-lactoglobulin–induced allergic mouse model. J Dairy Sci (2019) 102(3):1943–58. doi: 10.3168/jds.2018-15683 30660420

[16] Fu LL Xie MH Wang C Qian Y Huang JJ Sun ZH. *Lactobacillus casei* zhang alleviates shrimp tropomyosin-induced food allergy by switching antibody isotypes through the NF-kappaB-dependent immune tolerance. Mol Nutr Food Res (2020) 64(10):e1900496. doi: 10.1002/mnfr.201900496 32243079

[17] Bashir ME Andersen P Fuss IJ Shi HN Nagler-Anderson C. An enteric helminth infection protects against an allergic response to dietary antigen. J Immunol (2002) 169(6):3284–92. doi: 10.4049/jimmunol.169.6.3284 12218148

[18] Kawakita A Shirasaki H Yasutomi M Tokuriki S Mayumi M Naiki H. Immunotherapy with oligomannose-coated liposomes ameliorates allergic symptoms in a murine food allergy model. Allergy (2012) 67(3):371–9. doi: 10.1111/j.1398-9995.2011.02777.x 22423374

[19] Chakir H Wang H Lefebvre DE Webb J. Scott FW. T-bet/GATA-3 ratio as a measure of the Th1/Th2 cytokine profile in mixed cell populations: Predominant role of GATA-3. J Immunol Methods (2003) 278(1-2):157–69. doi: 10.1016/s0022-1759(03)00200-x 12957404

[20] Daynes RA Jones DC. Emerging roles of ppars in inflammation and immunity. Nat Rev Immunol (2002) 2(10):748–59. doi: 10.1038/nri912 12360213

[21] Coffman RL Savelkoul HF Lebman DA. Cytokine regulation of immunoglobulin isotype switching and expression. Semin Immunol (1989) 1(1):55–63. doi: 10.1016/0921-4488(91)90087-7 15630959

[22] Xiang QH Wu X Pan Y Wang L Cui CB Guo YW. Early-life intervention using fecal microbiota combined with probiotics promotes gut microbiota maturation, regulates immune system development, and alleviates weaning stress in piglets. Int J Mol Sci (2020) 21(2):503. doi: 10.3390/ijms21020503 PMC7014131 31941102

[23] Liao SF Nyachoti M. Using probiotics to improve swine gut health and nutrient utilization. Anim Nutr (2017) 3(4):331–43. doi: 10.1016/j.aninu.2017.06.007 PMC5941265 29767089

[24] Fujii M Fukuura K Ohto N Kuwahara H Mizuno M. *Lactobacillus plantarum* 22A-3 exerts anti-allergic activity through TGF-β secretion in passive cutaneous anaphylaxis of mice. Int J Food Sci Nutr (2020) 72(4):478–84. doi: 10.1080/09637486.2020.1833316 33076718

[25] Cella M Scheidegger D Palmer-Lehmann K Lane P Lanzavecchia A Alber G. Ligation of CD40 on dendritic cells triggers production of high levels of interleukin-12 and enhances T cell stimulatory capacity: T-T help *via* APC activation. J Exp Med (1996) 184(2):747–52. doi: 10.1084/jem.184.2.747 PMC2192696 8760829

[26] Chambers CA Allison JP. Co-Stimulation in T cell responses. Curr Opin Immunol (1997) 9(3):396–404. doi: 10.1016/s0952-7915(97)80087-8 9203422

[27] Abdel-Hamid M Romeih E Huang Z Enomoto T Huang L Li L. Bioactive properties of probiotic set-yogurt supplemented with siraitia grosvenorii fruit extract. Food Chem (2020) 303:125400. doi: 10.1016/j.foodchem.2019.125400 31470275

[28] Chen YQ Shi HZ. CD28/CTLA-4–CD80/CD86 and ICOS–B7RP-1 costimulatory pathway in bronchial asthma. Allergy (2006) 61(1):15–26. doi: 10.1111/j.1398-9995.2006.01008.x 16364152

[29] Wu ZG Rothwell L Young JR Kaufman J Butter C Kaiser P. Generation and characterization of chicken bone marrow-derived dendritic cells. Immunology (2010) 129(1):133–45. doi: 10.1111/j.1365-2567.2009.03129.x PMC2807494 19909375

[30] Joffre O Nolte MA Spörri R Sousa C. Inflammatory signals in dendritic cell activation and the induction of adaptive immunity. Immunol Rev (2009) 227(1):234–47. doi: 10.1111/j.1600-065X.2008.00718.x 19120488

[31] Huang CH Shen CC Lian YC Jan TR. The probiotic activity of *Lactobacillus murinus* against food allergy. J Funct Foods (2016) 25:231–41. doi: 10.1016/j.jff.2016.06.006

[32] Ma J Zhang J Li Q Shi Z Wu H Zhang H. Oral administration of a mixture of probiotics protects against food allergy *via* induction of CD103 + dendritic cells and modulates the intestinal microbiota. J Funct Foods (2019) 55:65–75. doi: 10.1016/j.jff.2019.02.010

[33] Jiang A Bloom O Ono S Cui WG Unternaehter J Jiang S. Disruption of e-cadherin-mediated adhesion induces a functionally distinct pathway of dendritic cell maturation. Immunity (2007) 27(4):610–24. doi: 10.1016/j.immuni.2007.08.015 PMC2151979 17936032

[34] Sica A Saccani A Bottazzi B Polentarutti N Vecchi A Damme JV. Autocrine production of IL-10 mediates defective IL-12 production and NF- kappaB activation in tumor-associated macrophages. J Immunol (2000) 164(2):762–7. doi: 10.4049/jimmunol.164.2.762 10623821

[35] Bashir MEH Louie S Shi HN Nagler-Anderson C. Toll-like receptor 4 signaling by intestinal microbes influences susceptibility to food allergy. J Immunol (2004) 172(11):6978–87. doi: 10.4049/jimmunol.172.11.6978 15153518

[36] Gertie JA Zhang BY Liu EG Hoyt LR Yin XY Xu L. Oral anaphylaxis to peanut in a mouse model is associated with gut permeability but not with Tlr4 or Dock8 mutations. J Allergy Clin Immun (2021) 149(1):262–74. doi: 10.1016/j.jaci.2021.05.015 PMC8626534 34051223

[37] Berin MC Zheng Y Domaradzki M Li XM Sampson HA. Role of TLR4 in allergic sensitization to food proteins in mice. Allergy (2010) 61(1):64–71. doi: 10.1111/j.1398-9995.2006.01012.x 16364158

